# Mixed neuroendocrine–non‐neuroendocrine neoplasm arising from long‐segment Barrettʼs esophagus showing exceptionally aggressive clinical behavior

**DOI:** 10.1002/cnr2.1644

**Published:** 2022-07-08

**Authors:** Kazuya Miyaguchi, Tomonori Kawasaki, Tomoaki Tashima, Shomei Ryozawa

**Affiliations:** ^1^ Department of Gastroenterology Saitama Medical University International Medical Center Saitama Japan; ^2^ Department of Pathology Saitama Medical University International Medical Center Saitama Japan

**Keywords:** esophagus, long‐segment Barrett's esophagus, mixed adenoneuroendocrine carcinoma, mixed neuroendocrine‐non‐neuroendocrine neoplasm, neuroendocrine carcinoma, neuroendocrine neoplasm

## Abstract

**Background:**

There is only one report of Barrett's esophagus (BE) with mixed neuroendocrine–non‐neuroendocrine neoplasm (MiNEN). Herein, for the first time, we present a case with an aggressive esophageal MiNEN, as well as with both primary MiNEN and conventional adenocarcinoma, arising in BE.

**Case:**

A 68‐year‐old woman had been diagnosed with 0‐IIa type adenocarcinoma in the background of long‐segment BE, 45 months earlier. She underwent endoscopic submucosal dissection (ESD) and the pathological diagnosis was tubular adenocarcinoma, well‐differentiated, with slight submucosal invasion. There was no lymphovascular invasion and the margins were intact. The upper esophagogastroduodenoscopy conducted the year after ESD showed no residual or recurrent cancer. However, she was subsequently followed up at another hospital, and endoscopy was not performed after the second year. She was urgently transported to our hospital due to buttock pain in the ninth month of the fourth year. A computed tomography (CT) of the head showed multiple cerebral metastases and positron emission tomography–CT revealed numerous osseous and nodal involvements. We performed upper endoscopy and detected type 3 esophageal tumor. Multiple biopsy specimens histopathologically contained invasive neoplasm composed of neuroendocrine carcinoma (NEC) and adenocarcinoma, moderately to poorly differentiated. The NEC element showed diffuse proliferation of primitive cancer cells possessing fine‐granular cytoplasm and nuclei with prominent nucleoli, whereas the adenocarcinoma component had tubules or nested growth of basophilic cells. Immunohistochemically, the NEC cells were diffusely positive for synaptophysin, with focal expressions of INSM1, chromogranin A and NCAM, whereas the adenocarcinoma cells were mostly negative for these NE markers. The Ki67 index was 90% at the hot spots in both types. The patient died 3.5 months after the biopsy‐based histological diagnosis.

**Conclusion:**

Appropriate therapy according to the guidelines and/or meticulous clinical follow‐up based on periodic endoscopy as well as a full physical examination are essential, from a proactive perspective, for early diagnosis of secondary aggressive cancers after ESD.

## 1 INTRODUCTION

The 2019 World Health Organization (WHO) classification defines mixed neuroendocrine‐non‐neuroendocrine neoplasm (MiNEN), so‐called mixed adenoneuroendocrine carcinoma (MANEC), as a combination of NE and non‐NE tumors with each of these components comprising at least 30% of the tumor.^1^ In the esophageal oncology field, there is only one report to the best of our knowledge, that by Kawazoe et al., of Barrett's esophagus with definite MiNEN/MANEC.^2^ Although Veits et al. also described Barrett's MANEC, the adenocarcinoma element accounted for only 20% of the total tumor area.^3^ Herein, for the first time, we present a case with an aggressive esophageal MiNEN, as well as with both primary MiNEN and conventional adenocarcinoma, arising in a long‐segment Barrett's esophagus (LSBE).

## 2 CASE PRESENTATION

**FIGURE 1:**
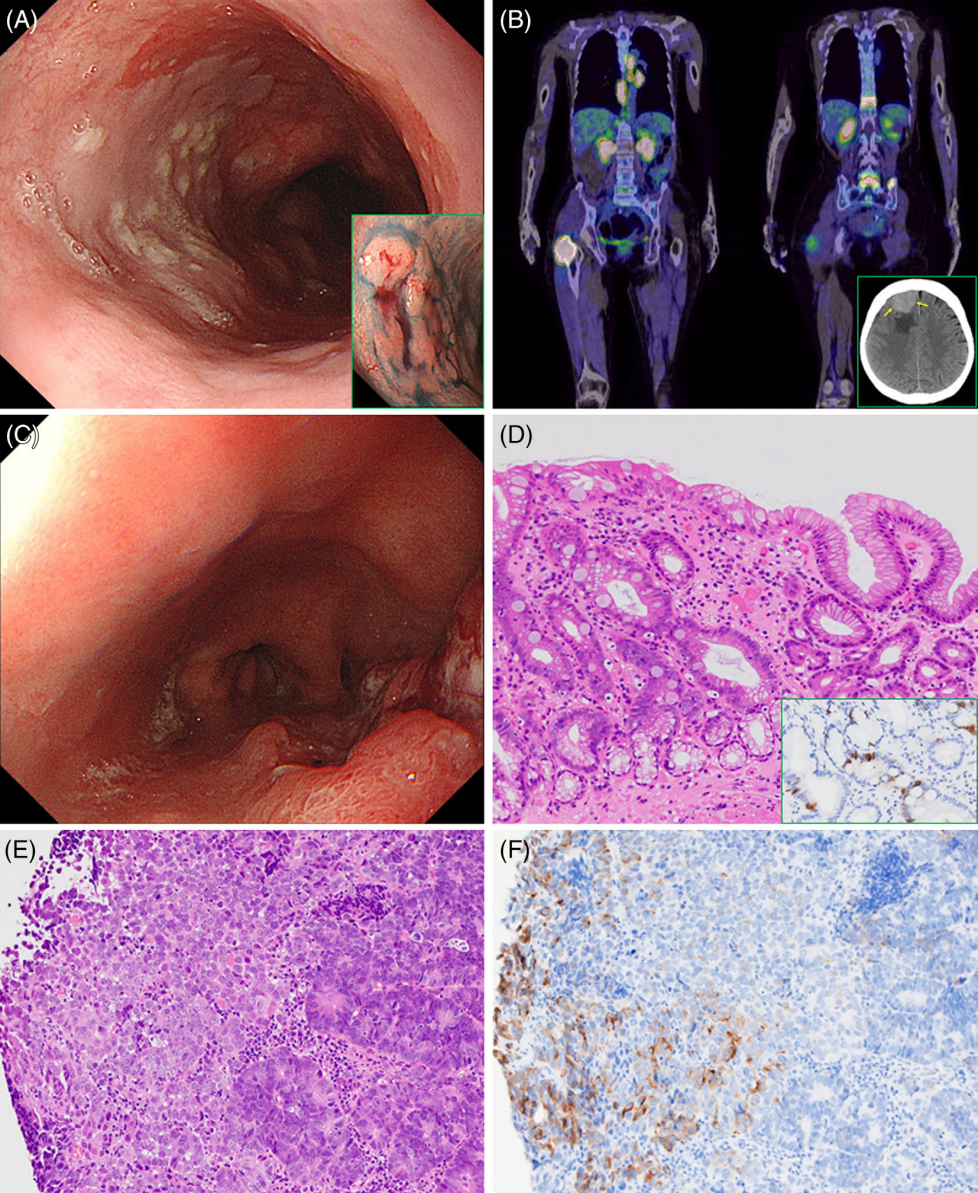
Clinicopathological findings of metachronous carcinomas derived from long‐segment Barrettʼs esophagus (LSBE). (A) LSBE spreading distally to 20 cm from the incisor teeth on upper endoscopy, with type 0‐IIa tumor (tubular adenocarcinoma) at the 23 cm incisor (inset). (B) Positron emission tomography–CT reveals multiple osseous metastases (11th thoracic vertebra, 5th lumbar vertebra and bilateral iliac bones), accompanied by osteolytic masses in the spinous process of the 5th lumbar vertebra and the proximal part of the right femur. Furthermore, left supraclavicular fossa (not included in this image), mediastinal, and paraaortic lymph nodes also show radioactive tracer accumulation. Inset: site of tumor location in the right frontal lobe on a CT slice (yellow arrows). (C) Advanced type 3 cancer, located 35 cm from the incisor, in the LSBE. (D) LSBE histologically possessing foveolar (right side) as well as metaplastic intestinal epithelia (left side) (H&E), with scattered chromogranin A‐immuno‐positive neuroendocrine cells (inset). (E) On biopsy sections, the type 3 esophageal tumor is comprised of an admixture of moderately to poorly differentiated adenocarcinoma (right side) and undifferentiated components (left side) (H&E). (F) Synaptophysin staining immunohistochemically demonstrates the neuroendocrine nature of the pattern‐less neoplastic region (left side)

A 68‐year‐old Japanese woman had been diagnosed with superficial elevated (0‐IIa) type adenocarcinoma in the background of LSBE (Figure 1A), measuring at 23 cm and 20 cm from the incisor teeth, respectively, 45 months earlier (in February 2017), at age 65. The TNM stage diagnosis of cT1N0M0, Stage I, was made using endoscopic ultrasonography and systemic computed tomography (CT). She underwent endoscopic submucosal dissection (ESD) at Saitama Medical University International Medical Center and the pathological diagnosis was tubular adenocarcinoma, well differentiated, 30 × 25 mm in size, with submucosal invasion (maximal depth: 133 μm, pT1b). There was no lymphovascular invasion and the margins were intact. Additional treatments, such as surgery and/or chemoradiotherapy, were recommended in accordance with the established guidelines. The patient was provided with fully informed consent regarding these options, but declined all procedures.

The upper esophagogastroduodenoscopy conducted the year after ESD showed no residual, recurrent, or new primary malignancies. The patient was subsequently followed up at another hospital, but endoscopy was not performed in the second and third years. She was brought to the emergency room by ambulance due to pain in the right hip after a fall, in November 2020. CT revealed a right femoral fracture. One day after emergent hospitalization, she developed seizures. A CT scan of the head revealed multiple brain metastases and positron emission tomography–CT showed numerous bone and nodal involvements (Figure 1B). We performed upper gastrointestinal endoscopy and detected an infiltrative ulcerative (Type 3) esophageal cancer at 35 cm from the incisors (Figure 1C).

Multiple biopsy specimens histopathologically contained invasive neoplasm composed of neuroendocrine carcinoma (NEC) and adenocarcinoma, moderately to poorly differentiated, accompanied by non‐neoplastic stomach‐like mucosa with intestinal metaplasia (Figure 1D,E). The NEC element showed diffuse proliferation of primitive cancer cells possessing fine‐granular cytoplasm and nuclei with prominent nucleoli, whereas the adenocarcinoma component had tubules or nested growth of basophilic cells due to a high nuclear/cytoplasmic ratio and hyperchromatic nuclei. On immunohistochemical examinations, the NEC cells were diffusely positive for synaptophysin, with focal expressions of insulinoma‐associated protein 1, chromogranin A, and CD56 (neural cell adhesion molecule), whereas the adenocarcinoma cells were mostly negative for these NE markers (Figure 1F). The Ki67 (MIB‐1) labeling index was very high (90% at the hot spots in both types). Based on these pathological features, this neoplasm was suggested to be an esophageal MiNEN (MANEC).

Radiotherapy (30 Gy/10 Fr) was subsequently administered for multiple cerebral metastases. The patient was transferred to a hospital for recommended palliative care and died 3.5 months after the biopsy‐based histological diagnosis.

## 3 DISCUSSION

It is generally recommended that the histopathological diagnosis be evaluated based on the entire tumor. However, unfortunately, in our current case, the diagnosis of MiNEN could only be made employing endoscopic biopsy samples, due to the rapidly deteriorating clinical course. The histological features of the deeper part of the tumor as well as the metastatic foci were, accordingly, not determined.

Esophageal NENs are considered to usually be derived from multipotential stem cells as well as Merkel cells in squamous epithelia.^4^ On the other hand, it is well known that gastric NEC and MiNEN/MANEC can arise from adenocarcinoma due to dedifferentiation or divergent differentiation rather than from existing enterochromaffin‐like cells, serving as the genesis of neuroendocrine tumors (so‐called carcinoids), or neuroendocrine cell micronests.^5^ In the current case, although LSBE had normal‐appearing, isolated/scattered neuroendocrine cells showing distinct chromogranin A and synaptophysin positivity, resembling proper gastric glands, MiNEN was composed of a mosaic admixture of adenocarcinoma and NEC with predominant synaptophysin immunoexpression, without the characteristic staining pattern of cytokeratin 20 (data not shown) as a Merkel cell marker. It is, therefore, reasonable to suggest that the NEN in our present case would most likely have originated from relatively primitive adenocarcinoma or Barrett's epithelium with pluripotentiality.^6^

Gurzu et al. investigated 13 gastrointestinal MANECs and demonstrated all to be microsatellite stable status (MSS) tumors.^7^ Our current MiNEN case also presented a proficient mismatch repair proteins (MMR) status with MLH1, MSH2, MSH6 and PMS2 immuno‐expressions (data not shown) in both the adenocarcinoma and the NEC elements, and these profiles were considered to be equivalent to MSS. In addition, Genitsch et al. studied tumor samples from 465 primary resected gastroesophageal (118 esophageal, 73 junctional, and 274 gastric) adenocarcinomas and none (0/118) of the esophageal adenocarcinomas were positive for Epstein–Barr (EB) virus‐encoded small RNAs by in situ hybridization,^8^ suggesting nonparticipation of the EB virus in carcinogenesis, as with our current case (data not shown).

Patients with MANEC in the esophagus are reportedly more likely to be diagnosed at an earlier stage and have significantly longer survival than those with pure NEC (median survivals of 28 and 15 months, respectively. *p* = 0.031).^9^ In fact, a previously reported patient with MANEC associated with LSBE, undergoing distal esophagectomy and proximal gastrectomy with lymph node dissection, was finally assessed as having Stage I (pT1, pN0, cM0), according to the eighth edition of the Union for International Cancer Control classification, with a good clinical course.^2^ Conversely, the Barrett's MiNEN in our current case had extremely aggressive biological behavior with antecedent distant metastases and rapidly progressed to a fatal outcome.

## 4 CONCLUSION

It should be kept in mind that esophageal MiNEN can occur not only in BE but also produce a very rapidly aggressive clinical course. While appropriate therapy according to the guidelines is highly recommended, meticulous clinical follow‐up based on periodic endoscopy as well as a full physical examination is, at a minimum, essential for high‐risk patients.

## AUTHOR CONTRIBUTIONS

**Kazuya Miyaguchi:** Conceptualization (equal); data curation (equal); formal analysis (equal); investigation (equal); methodology (equal); writing – original draft (equal). **Tomonori Kawasaki:** Conceptualization (equal); data curation (equal); formal analysis (equal); funding acquisition (lead); investigation (equal); methodology (equal); project administration (equal); resources (equal); software (equal); supervision (equal); validation (equal); visualization (equal); writing – original draft (equal); writing – review and editing (equal). **Tomoaki Tashima:** Conceptualization (equal); formal analysis (equal); investigation (equal); methodology (equal); project administration (equal); supervision (equal); validation (equal); visualization (equal). **Shomei Ryozawa:** Project administration (equal); supervision (equal); writing – review and editing (equal).

## Data Availability

The data that support the findings of this study are available on request from the corresponding author. The data are not publicly available due to privacy or ethical restrictions.
